# Antiphospholipid Syndrome-Related Pulmonary Embolism: Clinical Characteristics and Early Recognition

**DOI:** 10.3389/fcvm.2022.872523

**Published:** 2022-07-11

**Authors:** Maojing Shi, Weibo Gao, Yuebo Jin, Jihong Zhu, Yuansheng Liu, Tianbing Wang, Chun Li

**Affiliations:** ^1^Trauma Center, Peking University People's Hospital, Beijing, China; ^2^Department of Emergency, Peking University People's Hospital, Beijing, China; ^3^Department of Rheumatology and Immunology, Peking University People's Hospital, Beijing, China

**Keywords:** pulmonary embolism, antiphospholipid syndrome, antiphospholipid antibody, risk factors, score model

## Abstract

**Background:**

Pulmonary thromboembolism is a common disease frequently encountered in the emergency room and has a high mortality rate. Antiphospholipid syndrome (APS) is a high-risk factor for recurrent pulmonary embolism (PE). It is critical to effectively administer anticoagulants to avoid the recurrence of thrombotic events. This study aims to identify the clinical characteristics of APS patients with PE (APS-PE) and to develop a risk score for determining the presence of APS in PE patients in the emergency situations.

**Methods:**

We retrospectively enrolled 76 PE patients in this study, with 46 patients in the APS-PE group and 30 patients in the non-APS-PE group. We compared differences in demographics, laboratory parameters, and early mortality risk between the two groups. Risk factors for APS-PE were screened using logistic regression analysis. We also developed an early risk score using multivariate analysis weighted points proportional to the β*-* regression coefficient values and calculated the sensitivity and specificity for APS in PE patients.

**Results:**

In the APS-PE group, we observed a higher proportion of males (43.6 vs. 20%), a higher proportion of low-risk patients (58.7 vs. 10%), lower levels of white blood cells and platelets (PLT), longer activated partial thromboplastin time (APTT), and a slight increase in D-dimer levels. Patients who were triple positive for antiphospholipid antibodies (aPLs) were younger. The APTT gradually increased as the number of positive aPLs increased. The risk factors for APS included male (OR = 5.565, 95% CI 1.176–26.341), decreased PLT (OR = 0.029, 95% CI 0.003–0.330), slightly increased D-dimer (OR = 0.089, 95% CI 0.019–0.426), and prolonged APTT (OR = 4.870, 95% CI 1.189–19.951). The risk score was named MPDA and included male, PLT, D-dimer and APTT, which can predict APS in PE patients with the AUC at 0.888 (95% CI 0.811–0.965).

**Conclusion:**

The risk factors for APS in PE patients are male, low PLT, prolonged APTT and slightly increased D-dimer. The MPDA is a quantitative scoring system which is highly suggestive of APS in PE patients.

## Introduction

Pulmonary embolism (PE) is a group of diseases or clinical syndromes in which various emboli block the pulmonary artery or its branches. In most clinical contexts, emboli refer to blood clots (1). The global incidence, disability rates, and fatality rates of PE are high (1). PE is often accompanied by deep vein thrombosis (DVT) of the lower extremities, and both are referred to as venous thromboembolism (VTE). In addition to anticoagulation and thrombolysis, the causes of PE should be quickly identified and treated to avoid recurrence. Antiphospholipid syndrome (APS) is one of the strongest risk factors for DVT, with an odds ratio (OR ≥ 10) (2).

APS is an autoimmune disease characterized by thrombosis, pregnancy complications, and persistently positive antiphospholipid antibodies (aPLs). APS affects the vessels of tissues and organs, including the arteries, veins, and capillaries. Of the thrombosis, DVT and cerebral artery infarction are the most common complications (3). The incidence of DVT in APS patients could reach 38.9%, while the incidence of PE in APS patients is 14.1% (3). APS can also cause pulmonary hypertension, acute respiratory distress syndrome and intra-alveolar hemorrhage. The incidence of thrombosis in Chinese APS patients was 75.4%, of which 40.1% were DVT, 23.8% were stroke, and 6.7% were PE (4). Any embolism event in these APS patients can be life-threatening. VTE patients with positive aPL have a significantly increased risk of thrombosis recurrence after discontinuation of anticoagulation therapy (5, 6). Different from other PE patients, APS-PE patients require long-term anticoagulation therapy and may need additional immunotherapy (7, 8). Additionally, identifying APS in its early stages, particularly the early recognition of catastrophic antiphospholipid syndrome (CAPS), allows for early treatment, and improve survival.

This study aims to investigate the risk factors of APS from PE and develop a risk score for early recognition of APS.

## Patients and Methods

### Study Population

A total of 192 patients who were diagnosed with their first episode of PE between June 2018 and September 2021 in Peking University People's Hospital were retrospectively enrolled in this study. All demographic characteristics as well as clinical, radiological and laboratory data were collected from their medical records. PE was confirmed in all patients by computed tomographic pulmonary angiography (CTPA). DVT was confirmed by vascular ultrasound of the lower extremities. The laboratory data, including complete blood count and blood coagulation tests, were obtained from samples drawn when the patients were admitted to the Department of Emergency, while aPLs and blood chemistry tests were drawn in the morning of the next day, after the patients had fasted.

**Figure 1:**
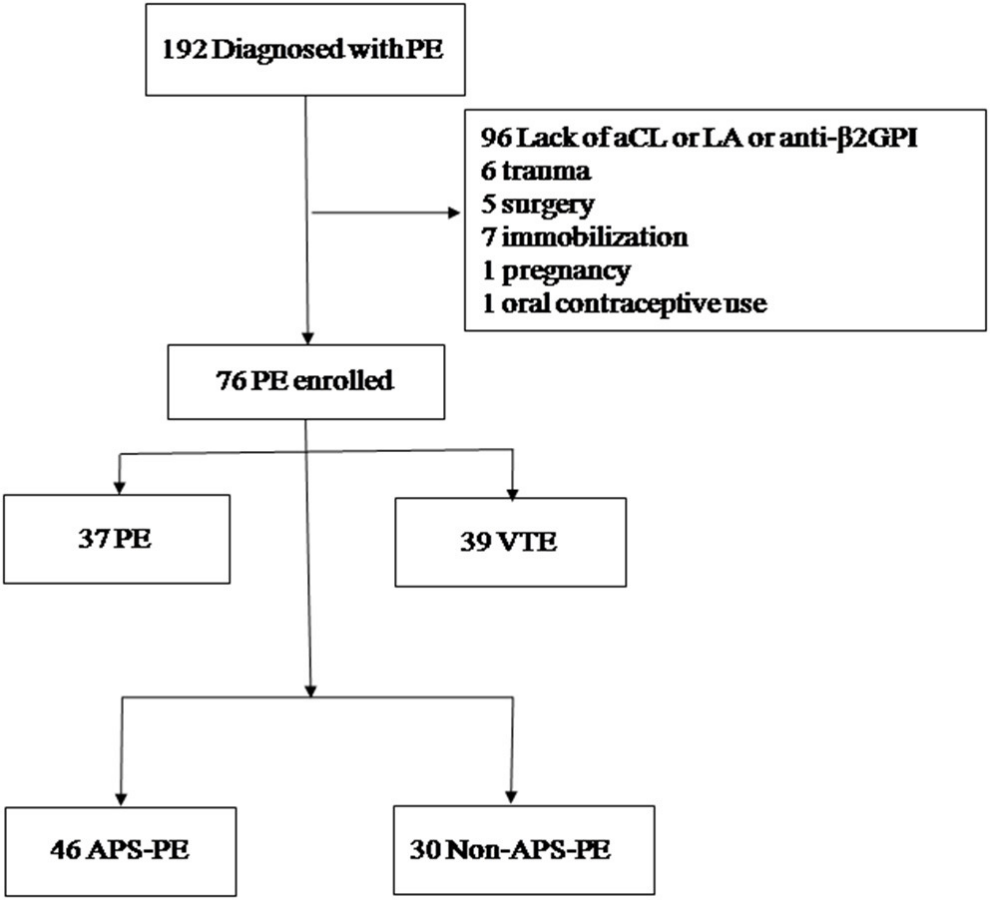
Patient selection flowchart. PE, pulmonary embolism; VTE, venous thromboembolism; DVT, deep vein thrombosis; aCL, anti-cardiolipin antibodies; LA, lupus anti-coagulant; aβ2GPI, anti-β2-glycoprotein I, APS, antiphospholipid syndrome.

APS was diagnosed according to the Sydney criteria (9). We tested for aPLs in PE patients repeatedly at least 12 weeks later. We excluded 96 patients who lacked aPLs results (Figure 1) and 20 PE patients who had other risk factors (e.g., trauma, surgery, immobilization, pregnancy, and oral contraceptive use) within the preceding 12 weeks. In total, 76 patients were included in this study. All patients experienced an initial episode of thrombosis. Forty-six patients were diagnosed with APS, and 30 patients were non-APS. In APS patients, 27 (58.7%) had primary APS (pAPS), 15 (32.6%) had systemic lupus erythematosus, and 1 (2.2%) patient presented with recurrent miscarriages.

The study was approved by the Institutional Review Board (IRB) of Peking University People's Hospital (No. 2022PHB007-001). Given the retrospective nature of this study, the requirement for informed consent was waived.

### Detection of aPLs

ELISA was used to identify IgA/IgG/IgM anti-β2-glycoprotein I (aβ2GPI) and IgA/IgG/IgM anti-cardiolipin antibodies (aCL), as previously described (10). Values for aCL > 12 IU/ml and aβ2GPI > 27 RU/ml were identified positive based on local cut-off. The simplified Dilute Russell's Viper Venom Test (dRVVT) was performed using Lupus anticoagulant (LA) 1 Screening reagent and LA2 Confirmatory reagent (STA, USA) according to the manufacturer's instructions. LA activity was considered positive when dRVVT ratios (LA1 screen/LA2 confirmation) were above 1.2 (11).

We calculated the adjusted global APS score (aGAPSS) by scoring the risk factors of each patient, as previously reported (12, 13). Five points were awarded for IgG/IgM aCL, 4 points were awarded for IgG/IgM aβ2GPI antibodies, 4 points for LA, 3 points for hyperlipidemia, and 1 point for arterial hypertension.

### Stratification

The pulmonary embolism severity index (PESI) correlates with the 30-day mortality of acute PE patients (14). Based on the PESI scores and other risk parameters (which accounted for hypertension and shock, right ventricular dysfunction observed during an imaging exam and cardiac laboratory biomarkers), the severity and risk of the early death of acute PE patients were stratified into high, intermediate-high, intermediate-low and low risk, according to the 2019 European Society of Cardiology (ESC) guidelines (15). Considering the complexity of the PESI, we used a simplified PESI (sPESI), which included the following variables: age, cancer, chronic cardiopulmonary disease, heart rate, systolic blood pressure and oxyhemoglobin saturation levels.

The 76 PE patients were divided into two groups based on whether they had APS: the APS-PE group (patients with a PE associated with APS) and the non-APS-PE group.

Seventy-six patients were divided into two groups: the PE group and the VTE group.

Patients were grouped as negative, single positive, double positive, or triple positive according to aPLs. Negative indicated the absence of LA, aCL or aβ2GPI. Single, double and triple positive indicated positivity of any one, two or all three aPLs.

### Statistical Analysis

Categorical variables were expressed as ratios and percentages of the total, while continuous variables were expressed as either the median and interquartile range (IQR) or the mean ± SD. The demographics, clinical and laboratory data were compared between PE patients with or without APS and patients with PE or VTE. A *t*-test or a Mann–Whitney *U* test was used to test the difference between parametric and non-parametric data between groups. A chi-square test or Fisher's exact test was used to assess categorical variables. One-way ANOVA or a Kruskal-Wallis test was used to compare the different positive antibody groups.

Age, gender and variables with *p* < 0.1 on univariate analysis were included in binary multivariate logistic regression analysis to identify the risk factors of APS. Risk factors were assigned based on points weighted by multivariate analysis. The β- regression coefficient values were divided by 1.583 (the lowest β- value) and rounded to the nearest integer. A linear transformation of the associated β-regression coefficient was used to score the risk factors (14, 16, 17); we then calculated the risk score for each patient. We also analyzed the areas under the receiver operating characteristic curve (AUC) of various risk factors to identify the sensitivity and specificity of these risk factors and risk scores. Statistical significance was identified using a two-sided *p* < 0.05; IBM SPSS Statistics version 22.0 (IBM Corp., Armonk, NY, USA) was used for all statistical analyses.

## Results

### Baseline Characteristics

Of the 76 subjects, 34.2% were males aged 56 ± 18 years old; 48.7% had PE without DVT, and 51.3% had VTE. Among all the subjects, the proportions of low risk, intermediate-low risk, intermediate-high risk and high risk were 39.5, 39.5, 15.8, and 5.2%, respectively. The positive rates of aCL, LA and aβ2GPI were 31.6, 53.9, and 27.6%, respectively. The positive rates of single, double or triple aPLs were 23.7, 21.1, and 15.8%, respectively. Of all the patients, 15.8% (*n* = 12) had thrombocytopenia (100 × 10^9^/L), 14.5% of patients belonged to the APS-PE group, and 1.3% belonged to the non-APS-PE group (Tables 1, 2).

**Table 1:** Demographic and clinical characteristics.

**Characteristics**	**Total (*n* = 76)**	**%**
Male	26	34.2%
Age, years	56 ± 18	
APS	46	60.5
Primary APS	27	58.7
Secondary APS	19	41.3
PE	37	48.7
VTE	39	51.3
Early mortality risk		
Low	30	39.5
Intermediate-low	30	39.5
Intermediate-high	12	15.8
High	4	5.2
aCL positive	24	31.6
aβ2GPI positive	21	27.6
LA positive	41	53.9
Single-positive	18	23.7
Double-positive	16	21.1
Triple-positive	12	15.8
PT, s	13.7 ± 4.7	
APTT, s	35.9 ± 13.8	
Fibrinogen, g/L	3.8 ± 1.3	
D-dimer, mg/L	1.8 (0.4, 2.9)	
WBC, × 10^9^/L	8.8 ± 4.0	
Hemoglobin, g/L	129.2 ± 24.1	
Platelet, × 10^9^/L	171.8 ± 85.9	
aGAPSS	4 (3, 10)	

*Data are presented as percentage (%), mean ± SD, or median (interquartile range) as appropriate*.

*PE, pulmonary embolism; VTE, venous thromboembolism; aCL, anti-caridolipin antibody; aβ2GPI, anti-β2-glycoprotein I antibody; LA, Lupus anticoagulant; PT, prothrombin time; APTT, activated partial thromboplastin time; WBC, white blood cell; aGAPSS, adjusted global antiphospholipid syndrome score*.

**Table 2:** Comparison of clinical characteristics between APS-PE and non-APS-PE patients.

**Characteristics**	**APS-PE** **(*n* = 46)**	**Non-APS-PE** **(*n* = 30)**	***p***
Age, years	53 ± 18	60 ± 17	0.090
Male, *n* (%)	20 (43.5%)	6 (20%)	0.035
Early mortality risk			
Low, *n* (%)	27 (58.7%)	3 (10%)	<0.001
Intermediate-low, *n* (%)	15 (32.6%)	15 (50%)	0.129
Intermediate-high, *n* (%)	2 (4.3%)	10 (33.3%)	0.002
High, *n* (%)	2 (4.3%)	2 (6.7%)	1.000
WBC, × 10^9^/L	8.0 ± 3.7	10.0 ± 4.3	0.043
Hemoglobin, g/L	126.6 ± 26.3	133.2 ± 20.2	0.261
Platelet, × 10^9^/L	154.8 ± 86.4	197.7 ± 79.9	0.036
PLT decrease(<100 × 10^9^/L)	11 (25.0%)	1(3.4%)	0.035
PT, s	14.4 ± 5.5	12.6 ± 3.2	0.084
APTT, s	40.3 ± 16.1	29.9 ± 6.5	0.001
Fibrinogen, g/L	3.7 ± 1.3	3.9 ± 1.2	0.461
D-dimer, mg/L	0.7 (0.2, 2.4)	2.6 (1.4, 3.8)	0.001
aCL(U/mL)	10.2 (4.0, 68.3)	1.8(3.1, 4.0)	<0.001
aβ2GPI(RU/mL)	15.2 (6.9, 64.7)	3.9(0.6, 6.1)	<0.001
LA	1.5 (1.3, 1.8)	1.1 (0.9, 1.1)	<0.001
Autoimmune disease	17 (37.0%)	5 (16.7%)	0.057
Antiplatelet therapy	4 (8.7%)	4 (13.3%)	0.794

*Data are presented as percentage (%), mean ± SD, or median (interquartile range) as appropriate*.

*APS, antiphospholipid syndrome; PE, pulmonaryembolism; WBC, white blood cell; PT, prothrombin time; APTT, activated partial thromboplastin time*.

### Comparison Between APS-PE and Non-APS-PE Patients

In the APS-PE group, there was a higher proportion of males (43.5 vs. 20%) and low-risk subjects (58.7 vs. 10%). The levels of white blood cells (WBC) and platelets (PLT) were lower in the APS-PE group (*p* < 0.05). Compared to the non-APS-PE group activated partial thromboplastin time (APTT) was significantly longer and the D-dimer level was significantly decreased in the APS-PE group (*p* < 0.05) (Table 2).

### Comparison Between the VTE Group and the PE Group

The VTE group had a higher proportion of males (51.3 vs. 16.2%) and APS patients (74.4 vs. 45.9%) (*p* < 0.05). There were no statistically significant differences between the two groups in terms of age, risk stratification, WBC, hemoglobin, PLT, APTT, D-dimer, the positivity of aPLs, or aGAPSS scores (Table 3).

**Table 3:** Comparison of clinical characteristics between the PE and VTE groups.

**Characteristics**	**PE (*n* = 37)**	**VTE (*n* = 39)**	***p***
Age, years	59 ± 16	53 ± 20	0.184
Male, *n* (%)	6 (16.2%)	20 (51.3%)	0.001
Positive antibodies		
Negative, *n* (%)	20 (54.1%)	10 (25.6%)	0.011
Single positive, *n* (%)	5 (13.5%)	13 (33.3%)	0.042
Double positive, *n* (%)	7 (18.9%)	9 (23.1%)	0.657
Triple positive, *n* (%)	5 (13.5%)	7 (17.9%)	0.596
APS-PE, *n* (%)	17 (45.9%)	29 (74.4%)	0.011
Early mortality risk		
Low, *n* (%)	13 (35.1%)	17 (43.6%)	0.451
Intermediate-low, *n* (%)	14 (37.8%)	16 (41%)	0.776
Intermediate-high, *n* (%)	9 (24.3%)	3 (7.7%)	0.062
High, *n* (%)	1 (2.7%)	3 (7.7%)	0.646
Laboratory findings		
WBC, × 10^9^/L	8.8 ± 3.3	8.8 ± 4.6	0.964
Hemoglobin, g/L	132.9 ± 21.9	126.7 ± 26.0	0.211
Platelet, × 10^9^/L	159.4 ± 76.0	184.0 ± 91.9	0.237
PT, s	14.0 ± 5.4	13.3 ± 3.9	0.525
APTT, s	33.1 ± 9.1	39.0 ± 17.3	0.083
Fibrinogen, g/L	3.8 ± 1.3	3.8 ± 1.2	0.848
D-dimer, mg/L	1.7 (0.2, 3.6)	1.9 (0.4, 2.6)	0.470
aGAPSS	4 (2, 10)	7 (4, 11)	0.205

*APS, antiphospholipid syndrome; PE, pulmonary embolism; VTE, venous thromboembolism; WBC, white blood cell; PT, prothrombin time; APTT, activated partial thromboplastin time; aGAPSS, adjusted global antiphospholipid syndrome score*.

### Clinical Characteristics Among Patients With Different aPL Profiles

Patients in the triple-positive group were younger than those in the other three groups. There was also a higher proportion of low-risk patients in the triple-positive group (*p* < 0.05). As the positive numbers of aPLs increased, the average APTT levels gradually prolonged, and the APTT of the triple positive groups was significantly longer than the other 3 groups. An opposite trend was observed in the D-dimer level (*p* < 0.05) (Table 4).

**Table 4:** Comparison of clinical characteristics within positive antibody groups.

	**Negative**	**Single positive**	**Double positive**	**Triple positive**	***p***
	**(*n* = 30)**	**(*n* = 18)**	**(*n* = 16)**	**(*n* = 12)**	
Age, years	60 ± 17	57 ± 17	57 ± 15	41 ± 21	0.020^*^
Male, *n* (%)	6 (20%)	10 (55.6%)	6 (37.5%)	4 (33.3%)	0.092
Early mortality risk					
Low, *n* (%)	3 (10%)	10 (55.5%)	8 (50%)	9 (75%)	<0.001^**^
Intermediate-low, *n* (%)	15 (50%)	6 (33.3%)	6 (37.5%)	3 (25%)	0.424
Intermediate-high, *n* (%)	10 (33.3%)	1 (5.6%)	1 (6.3%)	0 (0%)	0.005^**^
High, *n* (%)	2 (6.7%)	1 (5.6%)	1 (6.3%)	0 (0%)	0.696
VTE, *n* (%)	10 (33.3%)	13 (72.2%)	9 (56.3%)	7 (58.3%)	0.060
Laboratory findings					
WBC, × 10^9^/L	10.0 ± 4.3	8.2 ± 4.3	8.3 ± 3.6	7.4 ± 3.2	0.223
Hemoglobin, g/L	133.2 ± 20.2	122.1 ± 29.8	134.7 ± 22.8	123.0 ± 24.7	0.280
Platelet, × 10^9^/L	197.7 ± 80.0	159.8.0 ± 101.7	166.5 ± 80.7	133.2 ± 71.2	0.141
PT, s	12.6 ± 3.2	13.3 ± 2.4	16.1 ± 8.1	13.7 ± 3.6	0.132
APTT, s	29.9 ± 6.5	35.1 ± 11.9	39.3 ± 12.6	49.4 ± 22.7	0.001^*^
Fibrinogen, g/L	3.7 (3.1, 4.7)	3.5 (2.9, 4.2)	3.7 (2.5, 3.9)	4.4 (2.43, 5.4)	0.430
D-dimer, mg/L	2.6 (1.4, 3.8)	2.0 (0.3,2.9)	0.5 (0.1, 2.4)	0.5 (0.2, 1.0)	0.003^#^

* *p < 0.05 (triple-positive group vs. negative-, single-, or double- positive antibody groups)*.

# *p < 0.05 (negative-positive antibody group vs. double- or triple- positive antibody groups)*.

** *p < 0.05 (negative-positive antibody group vs. single-, double- or triple- positive antibody groups)*.

*VTE, venous thromboembolism; WBC, white blood cell; PT, prothrombin time; APTT, activated partial thromboplastin time*.

### Risk Factors and Risk Scores for APS-PE

The risk factors for APS in PE were male (OR = 5.565, 95% CI 1.176–26.341, *p* = 0.030), PLT level (OR = 0.029, 95% CI 0.003–0.330, *p* = 0.004), D-dimer level (OR = 0.089, 95% CI 0.019–0.426, *p* = 0.002), and APTT level (OR = 4.870, 95% CI 1.189–19.951, *p* = 0.028). The largest Youden index was used to determine the cut-off value, and the cutoff values for PLT, D-dimer, and APTT were 110 × 10^9^/L, 0.9 mg/L, and 32.2 s, respectively (Tables 5, 6).

**Table 5:** Univariate andmultivariate logistic analysis of variables associated with APS-PE.

**Variables**	**Univariate analysis**		**Multivariate analysis**	
	**OR (95%CI)**	***P***	**OR (95%CI)**	***p***
Age	0.977 (0.951, 1.004)	0.093	1.029 (0.974, 1.086)	0.312
Male	3.077 (1.058, 8.950)	0.039	5.565 (1.176, 26.341)	0.030
**Early mortality risk**				
Low	1.000		1.000	
Intermediate-low	0.111 (0.028, 0.447)	0.002	0.836 (0.063, 11.166)	0.892
Intermediate-high	0.022 (0.003, 0.153)	<0.001	0.072 (0.003, 1.768)	0.107
High	0.111 (0.011, 1.102)	0.061	7.561 (0.084, 677.418)	0.378
WBC	0.883 (0.779, 1.000)	0.050	0.957 (0.777, 1.179)	0.683
Hemoglobin	0.988 (0.968, 1.009)	0.259	-	-
Platelet	0.994 (0.988, 1.000)	0.043	0.029 (0.003, 0.330)	0.004
PT	1.113 (0.966, 1.282)	0.139	-	-
APTT	1.102 (1.024, 1.186)	0.010	4.870 (1.189, 19.951)	0.028
Fibrinogen	0.999 (0.995, 1.002)	0.456	-	-
D-Dimer	1.000 (0.999, 1.000)	0.015	0.089 (0.019, 0.426)	0.002
aCL	1.282 (1.061, 1.550)	0.010	-^*^	-
anti-β2GPI	1.149 (1.030, 1.283)	0.013	-^*^	-
LA	23540.943 (115.420, 4801379.120)	<0.001	-^*^	-

* *The aPLs were not accessible in the emergency environment, so they were not included in multivariate regression*.

**Table 6:** Multivariate logistic analysis of independent risk factors associated with APS-PE.

**Variables**	**β**	MPDA^*^	**Wald**	***p***	**OR**	**95%CI**	
Male	1.717	1	4.683	0.030	5.565 (1.176, 26.341)	1.176	26.341
PLT (<110 × 10^9^/L)	3.531	2	8.157	0.004	0.029 (0.003, 0.330)	0.003	0.330
D-dimer (<0.9 mg/L)	2.421	2	9.166	0.002	0.089 (0.019, 0.426)	0.019	0.426
APTT (≥32.2 s)	1.583	1	4.843	0.028	4.870 (1.189, 19.951)	1.189	19.951

* *Risk factors were scored using a linear transformation of the corresponding β-regression coefficient, all β coefficient were divided by the coefficient for APTT, and rounded to the nearest integer. MPDA = 1 × Male + 2 × PLT + 2 × D-dimer+1 × APTT (in this formula, Males were assigned 1, PLT <110× 10^9^/L was assigned 1, D-dimer <0.9 mg/L was assigned 1, APTT ≥ 32.2s was assigned 1)*.

*OR, odds ratio; CI, confidence interval*.

**Figure 2:**
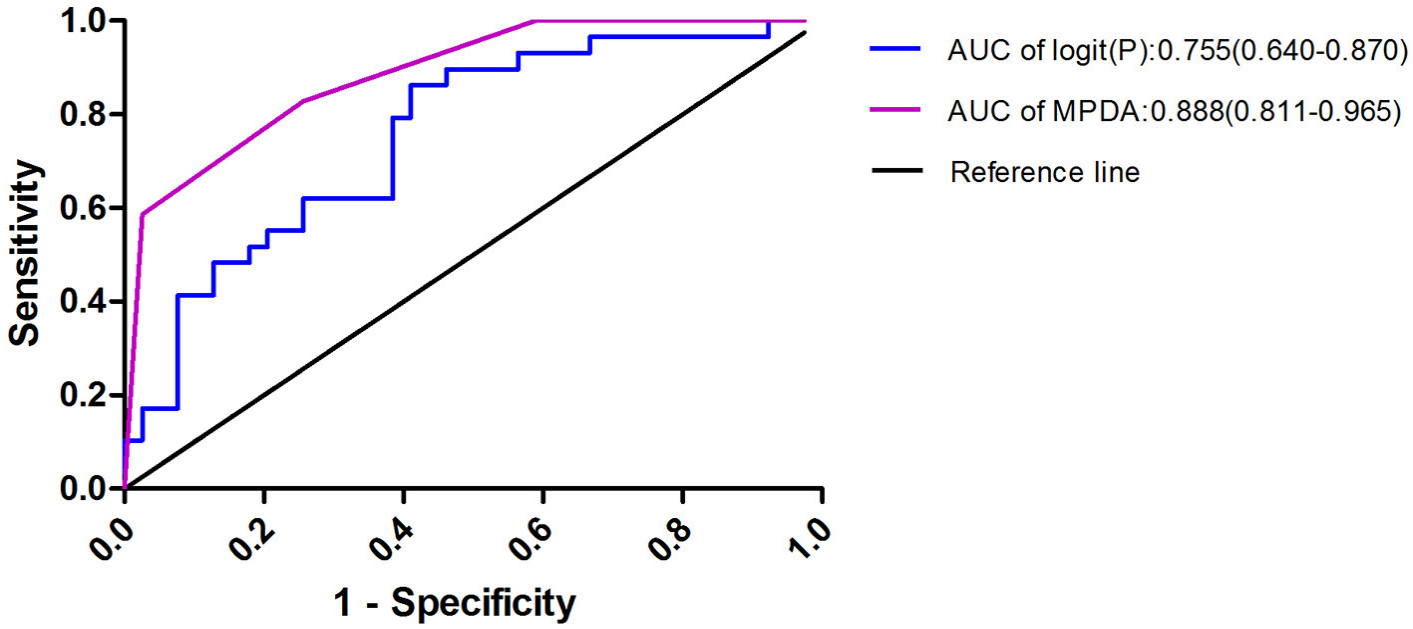
Risk factors were used to produce the receiver operating characteristic curves logit(P) = 1.717 × Male + 3,531 × PLT + 2.421 × D-dimer + 1.583 × APTT; MPDA = 1 × Male + 2 × PLT + 2 × D-dimer + 1 × APTT. When MPDA was more than 2, the predicted sensitivity of APS -PE was 84.6% and the specificity was 75.9%.

To facilitate the clinical application of the predictive model, the β- regression coefficient value (Model I) was rounded to the nearest integer (Model II, named MPDA) and used as the value of each risk factor; this scoring model was displayed in Table 6. Confirmed APS was considered the gold-standard, and the AUC of the two scoring models (Model I and MDPA) were 0.755 (95% CI 0.640–0.870) and 0.888 (95% CI 0.811–0.965). The MPDA had a higher AUC, demonstrating sufficient predictive value. The maximum Youden index (when the score was 2 points) was set as the cutoff value of MPDA, and the sensitivity was 84.6% and the specificity was 75.9% (Figure 2).

## Discussion

Our results demonstrated that there were more males, higher proportions of low-risk patients, lower WBC and PLT levels, prolonged APTT, and lower D-dimer levels in the APS-PE group. Therefore, the MPDA scoring system, based on gender, PLT, D-dimer, and APTT, could help healthcare professionals identify APS patients in emergency situations.

The average age of patients with primary APS who experienced their first thrombotic event was younger than 50 years old (18). The average age of the APS-PE group was 39.7 years, while the average age of the non-APS-PE group was 60.4 years (19), which could be related to the younger age of APS onset (4, 20). Therefore, aPLs should be tested in patients younger than 40 years old who first experienced symptomatic PE or in young patients (<50 years old) with unexplained thrombotic events at an abnormal site or in patients who suffered from pregnancy complications (19, 21, 22).

Compared to patients without thrombosis, there was a higher proportion of males in APS patients with thrombosis and arterial thrombosis complications (23). Our study was consistent with previous studies, which could be related to the sex hormones in young females as a protective factor for vascular endothelium (24). Therefore, APS screening should be considered when PE occurs in young males, especially when accompanied by DVT.

In this study, there were more low-risk patients observed in the APS-PE group, especially in triple positive aPLs patients. This was consistent with previous studies, which found 62.5% of low-risk patients in the APS-PE group and only 29.5% in the non-APS-PE group (19). Therefore, the aPLs test should not be ignored for patients with low-risk pulmonary embolism.

Thrombocytopenia is a common hematological manifestation of APS and occurrs in about 30% of APS patients (3). Among aPL carriers, patients with thrombocytopenia have a high risk of developing thrombosis (25–27). Platelet activation plays a key role in thrombocytopenia and thrombosis. Possible mechanisms are platelet activation and aggregation by aPL, and destruction of platelets by antibodies directed against platelet membrane glycoproteins (28). Platelets play a key role in the pro-thrombotic interaction between aPLs and endothelial cells (29). It has been reported that thrombocytopenia is a predictor of poor APS outcomes and is associated with an increased risk of CAPS and poor long-term survival (30, 31). Therefore, thrombocytopenia in APS patients might indicate more severe APS (including an increased risk of thrombosis) (26). Additionally, thrombocytopenia in APS is not a contraindication for anticoagulation.

Consistent with previous studies (19), prolonged APTT was an indicator of APS in young PE patients. The LA antibody may inhibit the formation of prothrombinase complexes, leading to prolonged APTT (32). Therefore, along with a history of thrombosis or pregnancy morbidity, mild thrombocytopenia and unexplained prolongation of APTT could indicate APS (33).

D-dimer is widely used as a marker for the hypercoagulable state in clinical practice. High D-dimer levels could assist in the diagnosis of venous thrombosis (34, 35). The diagnostic sensitivity of D-dimer to acute PE is high (80–100%), and its negative predictive value could reach 100% (36). The persistent elevation of D-dimer provides further information for predicting the recurrence risk and informing treatment decisions (37). The D-dimer level of the APS-PE group in this study is similar to another study conducted in China (38). Larger sample sizes are required for further research.

It has been proposed that although aPLs persistently present, thrombotic events occur only occasionally. This indicates that environmental factors (infection), inflammatory factors (concomitant autoimmune diseases), or other non-immune procoagulant factors (such as contraceptives containing estrogen, surgery, and immobility) are involved in the thrombosis process, which is known as the “two-hit model” (39). Two thrombosis prediction models, including aPL-S scores and the global APS score (GAPSS) were developed. The aPL-S score includes 3 lupus anticoagulant tests and 6 solid-phase antiphospholipid tests (40), among which a score ≥30 is defined as an independent risk factor for thrombosis (*p* = 0.006) (40). Among SLE patients, GAPSS ≥10 could be the best risk prediction for assessing the outcome of thrombosis and pregnancy loss (16). Among subjects with primary APS, those with thrombosis had higher GAPSS values than those without. The GAPSS value of patients with recurrence of thrombosis is higher than those without recurrence (20). GAPSS values ≥11 are the most reliable predictor in terms of sensitivity and specificity (20). Both models can be used to assess the risk of thrombosis in APS, but both scores were based on aPLs. Therefore, they cannot be used in the emergency situations. In this study, we proposed an APS prediction model based on conventional clinical indicators to identify APS as a risk factor for primary PE in the emergency room (ER). This score model is sensitive and specific, and the parameters involved are immediately available, which helps ER doctors who must make quick decisions.

## Limitations

The limitations of our study include its design as a single-center retrospective study and the relatively small size of the cohort. Many patients did not test for aPLs, and some patients only checked for one-time aPL. Therefore, several possible APS patients were not included in the study.

Further multicenter prospective studies are needed, and our proposed MPDA score (male, reduced PLT, increased D-dimer, and prolonged APTT) can provide ER physicians with a useful screening tool to manage APS. It can help to quickly identify potential APS-PE patients, assess the need for long-term anticoagulation therapy, and decrease the rate of recurrent thrombosis.

## Conclusions

In this study, we explored the independent predictors of APS-PE patients and established an MPDA score for predicting APS in PE patients. In this model, the most sensitive predictor of APS is male, reduced PLT, slightly prolonged D-dimer, and prolonged APTT. The MPDA score, which is based on the above four clinical indicators, provides a basis for quickly identifying these individuals in the ER. PE patients with these characteristics should be tested for aPLs and screened for APS.

## Data Availability Statement

The raw data supporting the conclusions of this article will be made available by the authors, without undue reservation.

## Ethics Statement

The studies involving human participants were reviewed and approved by Institutional Review Board (IRB) of Peking University People's Hospital (No. 2022PHB007-001). Written informed consent for participation was not required for this study in accordance with the national legislation and the institutional requirements.

## Author Contributions

MS, YJ, CL, and WG designed the study, analyzed the data, and drafted the manuscript. CL and JZ helped with statistical analysis, intellectual discussions, and editing. TW, YL, and CL provided critical suggestions for improving the manuscript. All authors contributed to the article and approved the submitted version.

## Funding

This study was supported by grants from the Beijing Natural Science Foundation (7192211) and the National Key R&D Program of China (2018YFF0301103).

## Conflict of Interest

The authors declare that the research was conducted in the absence of any commercial or financial relationships that could be construed as a potential conflict of interest.

## Publisher's Note

All claims expressed in this article are solely those of the authors and do not necessarily represent those of their affiliated organizations, or those of the publisher, the editors and the reviewers. Any product that may be evaluated in this article, or claim that may be made by its manufacturer, is not guaranteed or endorsed by the publisher.
